# “Nothing About Me Without Me”: An Interpretative Review of Patient Accessible Electronic Health Records

**DOI:** 10.2196/jmir.4446

**Published:** 2015-06-29

**Authors:** Sagar Ramesh Jilka, Ryan Callahan, Nick Sevdalis, Erik K Mayer, Ara Darzi

**Affiliations:** ^1^ Centre for Health Policy Department of Surgery and Cancer Imperial College London London United Kingdom; ^2^ Centre for Implementation Science Health Service & Population Research Department King’s College London London United Kingdom

**Keywords:** patient accessible electronic health records, online record access

## Abstract

**Background:**

Patient accessible electronic health records (PAEHRs) enable patients to access and manage personal clinical information that is made available to them by their health care providers (HCPs). It is thought that the shared management nature of medical record access improves patient outcomes and improves patient satisfaction. However, recent reviews have found that this is not the case. Furthermore, little research has focused on PAEHRs from the HCP viewpoint. HCPs include physicians, nurses, and service providers.

**Objective:**

We provide a systematic review of reviews of the impact of giving patients record access from both a patient and HCP point of view. The review covers a broad range of outcome measures, including patient safety, patient satisfaction, privacy and security, self-efficacy, and health outcome.

**Methods:**

A systematic search was conducted using Web of Science to identify review articles on the impact of PAEHRs. Our search was limited to English-language reviews published between January 2002 and November 2014. A total of 73 citations were retrieved from a series of Boolean search terms including “review*” with “patient access to records”. These reviews went through a novel scoring system analysis whereby we calculated how many positive outcomes were reported per every outcome measure investigated. This provided a way to quantify the impact of PAEHRs.

**Results:**

Ten reviews covering chronic patients (eg, diabetes and hypertension) and primary care patients, as well as HCPs were found but eight were included for the analysis of outcome measures. We found mixed outcomes across both patient and HCP groups, with approximately half of the reviews showing positive changes with record access. Patients believe that record access increases their perception of control; however, outcome measures thought to create psychological concerns (such as patient anxiety as a result of seeing their medical record) are still unanswered. Nurses are more likely than physicians to gain time efficiencies by using a PAEHR system with the main concern from physicians being the security of the PAEHRs.

**Conclusions:**

This review implements a novel scoring system, which shows there is a lack of rigorous empirical testing that separates the effect of record access from other existing disease management programs. Current research is too targeted within certain clinical groups’ needs, and although there are positive signs for the adoption of PAEHRs, there is currently insufficient evidence about the effect of PAEHRs on health outcomes for patients or HCPs.

## Introduction

Modern technology is changing the role of the passive patient to a more informed and engaging stakeholder in their own care [1]. Technology is making personal health-related data and documents digitally accessible and shareable between patients and physicians, with the aim of improving the safety, quality, and effectiveness of care [2]. According to the Council of Europe, patients should be in a position to access their medical records at their request and also be able to control who else can see their records [3]. Despite such calls, it is still not common practice for patients to access their medical records [4].

The use of patient accessible electronic health records (PAEHRs) has been considered by health organizations since the early 1990s [5]; however, PAEHRs have only recently received attention for their use in improving access to patient data [6,7]. In their early days, PAEHRs failed to gain approval for adoption because of prohibitive financial cost and the difficulty of transitioning from paper-based records [8]. With the advancements of modern technology, PAEHR systems should be technologically easier to implement and administer, yet the question still remains: Why has modern medicine not yet seen more widespread application and implementation of PAEHR in patient care?

One potential reason is that research has still not resolved whether patients want to access their medical records. Assuming patients would like access to their records, it is not yet known how helpful their medical record (in its typical current form) will be to them and whether patients will understand its content [1,4]. Furthermore, we currently have no knowledge of the impact that patient access to their PAEHRs would have on health care providers (HCPs) [9].

To date, research on the impact of PAEHRs has been focused on a particular clinical group, or on a limited number of outcome measures, from either the perspective of patients or doctors. Furthermore, no data have been published regarding the impact of changes in information supply—whether qualitative or quantitative—on patients’ psychological status, for example, their anxiety about their health [10]. To address the above issues, we provide a review of existing reviews that aims to critically evaluate the current state of the evidence regarding PAEHRs. The main objective of this paper was to synthesize relevant research to provide a quantitative insight into the impact of PAEHRs across a range of outcome measures in a number of clinical populations and investigate differences between patients and HCPs.

## Methods

### Study Search and Selection

We searched English-language articles indexed in any databases in Web of Science with a publication date between January 2002 and November 2014. Potentially relevant review articles were identified using a combination of medical subject headings, free text phrases, and Boolean searches. These included “review*” with (1) “patient access to records” (n=49 citations), (2) “patient portal” (n=18 citations), and (3) “patient accessible” (n=6 citations) across all Web of Science databases, including Web of Science core collection, MEDLINE, and BIOSIS Citation Index. This allowed us to focus the current paper as a review of reviews within the existing literature resulting in 73 citations. The references of selected reviews were also examined to search for additional articles satisfying inclusion criteria (n=1).

### Eligibility Criteria

**Figure 1:**
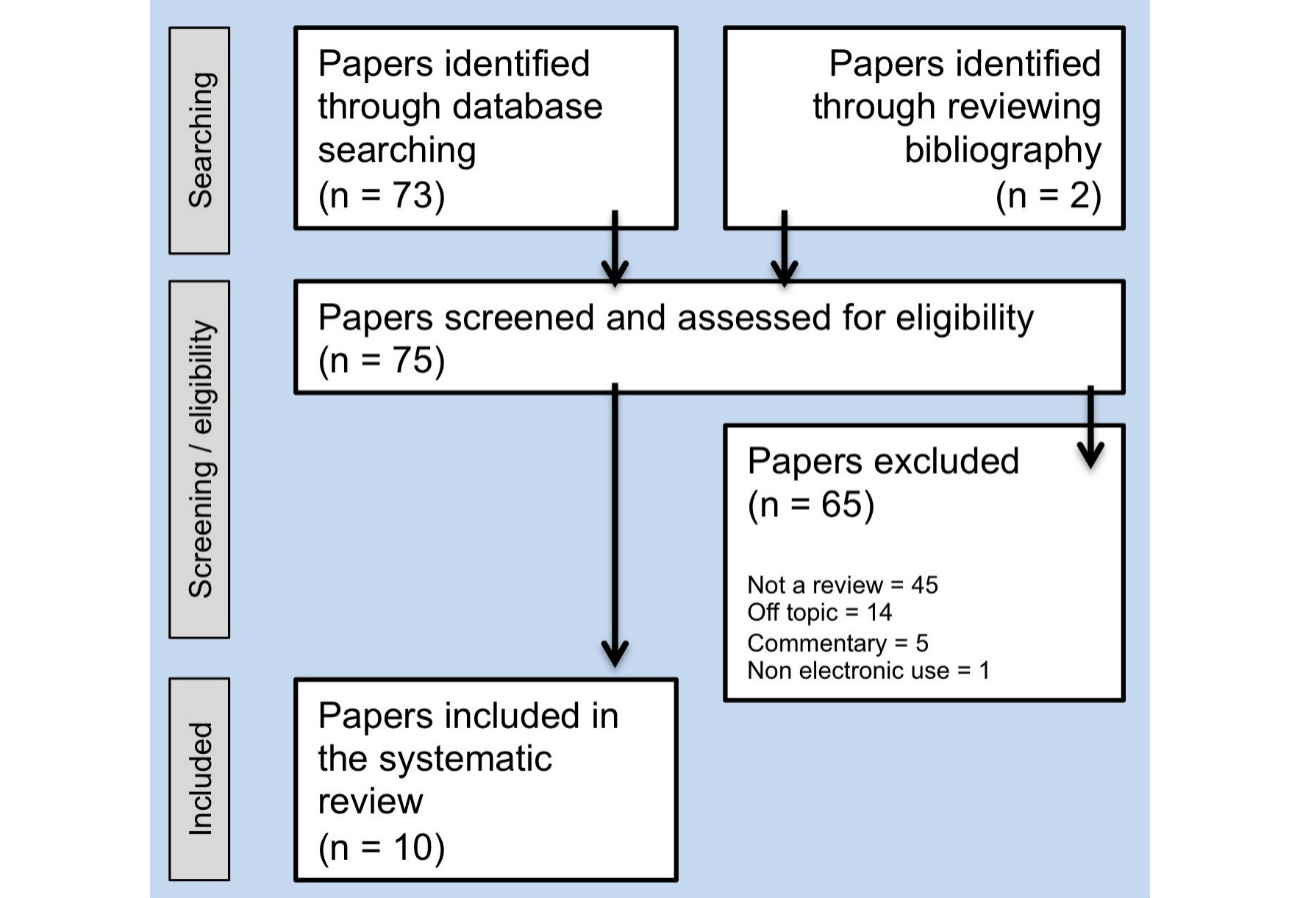
Flow diagram of study methodology.

We defined PAEHRs as patient accessible information held by the physician and/or health care system. We included systematic reviews that assessed the effect of PAEHRs on a variety of quality and clinically related outcome measures in adult populations. The reviews investigated patients suffering chronic disease such as diabetes and hypertension as well as patients seen in primary care. Inclusion criteria included suitable research questions, description of methods supporting the paper as a review, and reported a narrative on the impact of PAEHRs. Exclusion criteria were non-English, non-peer-reviewed, duplicates, non-empirical, and papers with a non-electronic use of record access or if the focus of the paper was on the design of a patient portal system. The majority of citations were excluded because they did not provide a review of the existing literature on patient/HCP outcome measures based on a review of the abstract and/or study title (Figure 1).

### Scoring System

A scoring system was developed to weight the impact of an outcome measure quantitatively and thereby investigate the impact of PAEHRs by individually assessing their impact on each outcome measure described in the reviews. These outcome measures were subsequently categorized forming 16 measurable domains (Multimedia Appendix 1). The definitions of these outcome measures are either (1) derived directly from one of the original review sources (eg, “effectiveness of record access” and “usefulness and usability” have been concatenated to make the definition “usefulness/effectiveness of record access”), or (2) a logical definition has been applied based on the original definitions (eg, “glycemic control, change in gyrated hemoglobin and blood pressure control” have been concatenated to make the definition “clinical outcome”). The citations of each included review were assessed to determine which outcome measures were investigated (frequency) as well as the result of that outcome measure, that is, if the investigated outcome measure was found to improve as a result of PAEHR access (positive impact). For example, in a review by Giardina et al [9], a study was included carried out by McCarrier et al, which evaluated the effectiveness of electronic patient portals in a group of diabetic patients [11]. McCarrier found that there was no improvement in glycemic control in patients with PAEHR access (clinical measure), but patients became more involved in their clinical care through the use of PAEHRs (self-efficacy - patient involvement), therefore providing a positive outcome score of 1 in the “self-efficacy - patient involvement” outcome measure and a no improvement score of 0 in the “clinical outcome” outcome measure.

## Results

### Overview

**Figure 2:**
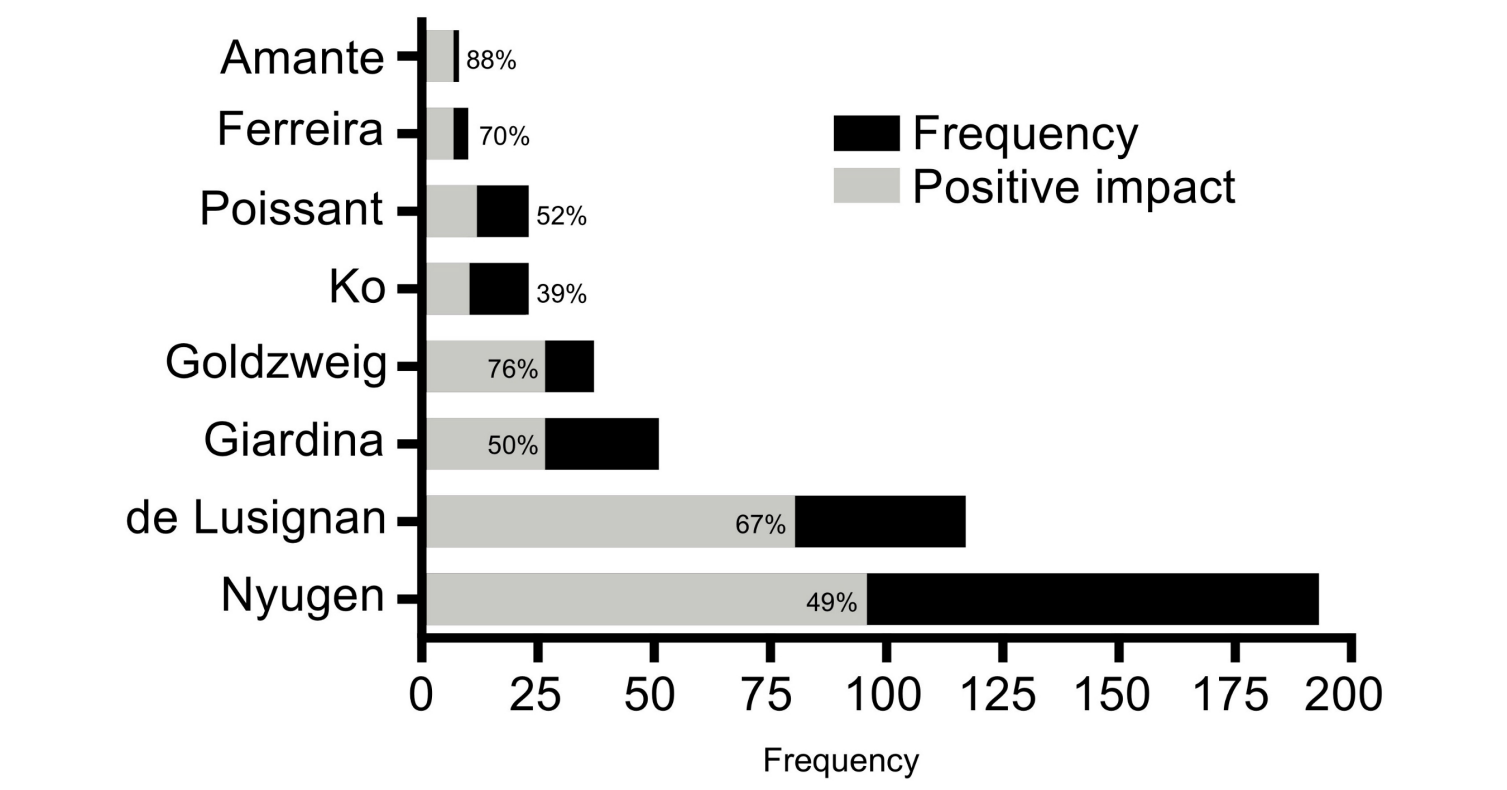
Frequency of all outcome measures across the 8 reviews analyzed in this study. The number of times a review (y-axis) reported on any outcome measure (black bar) against the number of times these outcome measures were found to have a positive impact (gray bar).

The systematic search provided ten review articles reporting on PAEHR implementations across different health care contexts and clinical groups (Multimedia Appendix 2; [12]). Eight review articles were used in the final analysis. One review was excluded because of duplicate citations [1], and another study [7] contained 32 citations that were not referenced directly within the outcome measures described in their paper. Figure 2 summarizes the total number of times an outcome measure was reported in each review against the number of times these outcome measures were reported to have had a positive impact across each individual study.

### Patients’ Perspective

**Figure 3:**
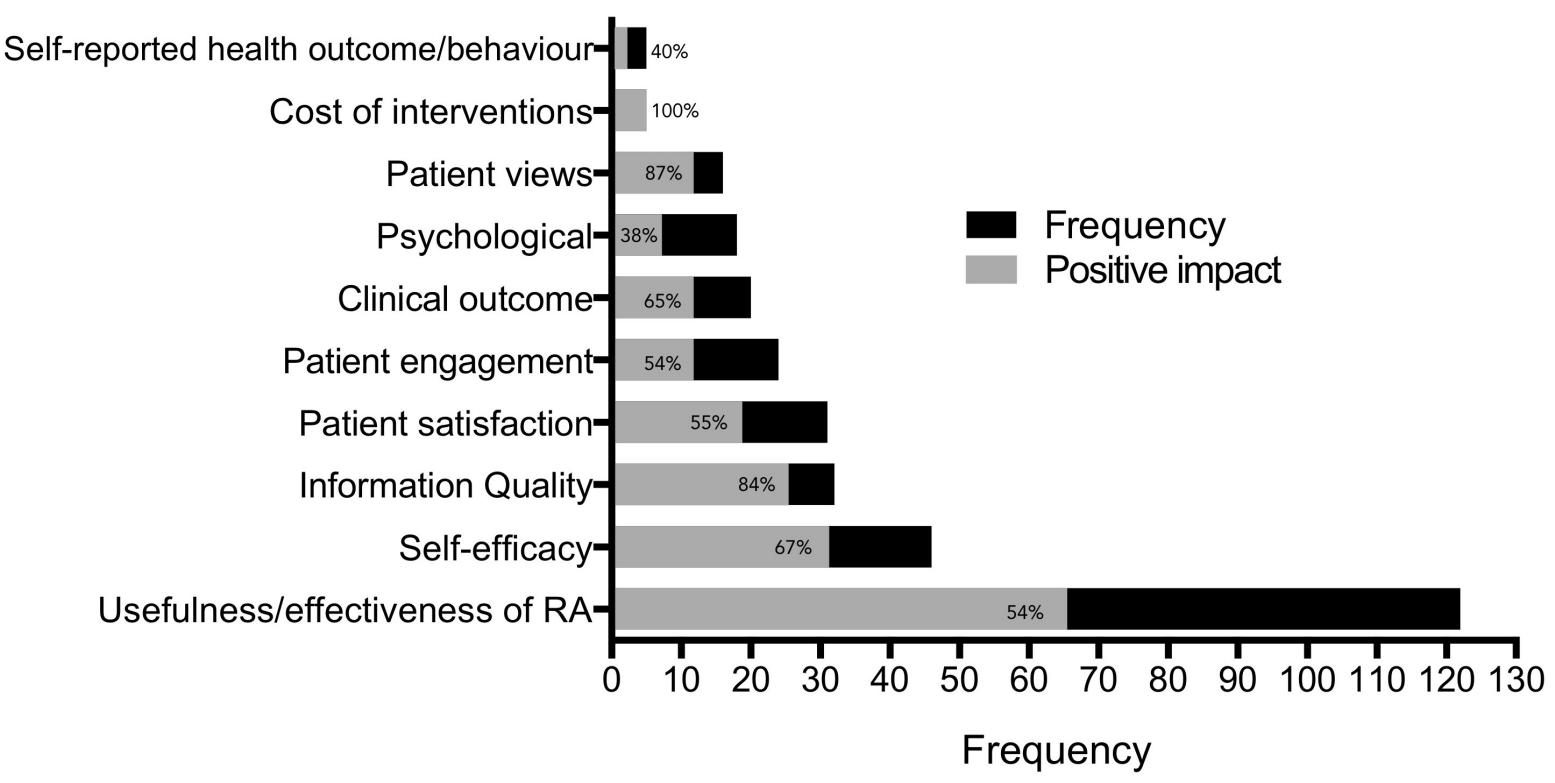
The frequency of patient outcomes (black bars) against frequency of positive change (gray bars); eg, usefulness/effective of record access (RA) has been investigated 122 times as an outcome measure with only 66 of those investigations reporting a positive impact (gray bar). We therefore infer that the proportion of black on the horizontal bars illustrates that there are studies that have found RA to have a negative impact or at least no impact on the outcome factors.

Across reviews, we found some uncertainty regarding whether access to PAEHRs makes a difference and whether patients actually want access to their PAEHRs (Figure 3).

#### Usefulness/Effectiveness of Patient Access to Electronic Health Records

The usefulness/effectiveness of PAEHRs included outcomes such as the usefulness, interoperability, and adoption rate. A more detailed list can be found in Multimedia Appendix 1. It is unclear from the current evidence whether PAEHRs are useful or effective for patients. Giardina found 40% (2/5) of studies showing positive outcomes of PAEHR usefulness [9], Nyugen found 50% (43/86), and Poissant found 53% (10/19). Two reviews found PAEHRs showed an overall positive impact: 100% for both de Lusignan (9/9) and Goldzweig (2/2). Whereas Ferreira found the opposite effect (0/1).

Nyugen et al reported that patients questioned the usefulness of PAEHRs because they were not well designed and did not integrate well with other existing clinical systems, for example, the National Health Service (NHS) HealthSpace [13]. Four themes emerged from the current review that act as a framework for usefulness: (1) promotion of a sense of illness ownership, (2) patient driven communication, (3) personalized support, and (4) mutual trust between patient and provider.

#### Patient Satisfaction

Patient satisfaction was investigated with outcomes such as mood states and satisfaction with care [14] and is further defined in Multimedia Appendix 1. We found six reviews that reported on patient satisfaction. Of those, two reviews [4,14] found no change in patient satisfaction (0/1 in both reviews) and one review reported 14% (1/7) that included showing a positive impact on patient satisfaction after PAEHR use [9]. Nyugen found 40% (2/5) of studies [13] and Goldzweig reported 57% (4/7) of studies showing a positive impact on patient satisfaction [15]. De Lusignan reported 100% (10/10) of studies showing a positive change in patient satisfaction [16].

A barrier to PAEHR uptake is poor patient satisfaction with a PAEHR system. Satisfaction can be a result of various aspects of patient experience, such as the (perceived) quality of care, consultation, or information provided [9]. Giardina et al found 11 studies that reported on patient satisfaction with eight of them showing no significant differences in satisfaction as a result of PAEHR access [9]. Similarly, Ferreira et al found that use of PAEHRs produces only modest benefits in satisfaction [4].

#### Patients’ Self-Efficacy

Self-efficacy involved various aspects that encompass a patient’s beliefs about how they feel, including patient involvement, communication, and patient empowerment as a result of PAEHR access. Overall, we found 67% (31/46) of positive changes as a result of PAEHR use across all self-efficacy domains, as made up by patient involvement (67%, 10/15), patient empowerment (78%, 18/23), and patient communication (38%, 3/8).

The most common reasons that patients wanted to look at their medical records were to see what their physician said about them (74%), to be more involved in their health care (74%), and to understand their condition better (72%) [4]. Ko et al report patient empowerment outcomes in 3 clinical groups, namely oncology (n=2), and palliative care (n=1) demonstrating positive change after PAEHR use, and a negative change in a group of rheumatoid arthritis patients (n=1), and two studies in oncology with patient communication as an outcome (both showing no change in communication with PAEHR access) [14].

#### Psychological Outcomes

Psychological outcomes examined across reviews included measures of anxiety, depression, contentment, and quality of life, using behavioral measures such as the Spielberger State-Trait Anxiety Inventory and the European Organization for Research and Cancer quality-of-life questionnaire (EORTC QLQ-C30) [17]. We found a typical pattern of mixed outcomes with 11 studies showing no change in psychological outcomes from a total of 18. For example, a study reported in Goldzweig et al randomly assigned couples having in vitro fertilization in the Netherlands to usual care versus PAEHR access and found no change in anxiety or change in depression between the 2 groups as a result of PAEHR access [18]. Poissant et al also report that PAEHR access was not found stressful by patients [19]. Ferreira et al found no consistent pattern in the impact of PAEHRs on psychological outcomes and suggested it is worthwhile to carry out a larger study on the effects of PAEHR use on such outcomes [4].

#### Health Outcomes/Behaviors and Clinical Outcomes

Health outcomes/behavior include diet, alcohol intake, medication changes, and smoking or exercise habits and are different to “clinical outcomes”, which refer to outcomes that can be empirically tested such as hemoglobin A1c levels. Giardina et al’s review shows a typical pattern of PAEHR impact, whereby they found a mix of results relating to specific clinical measures (such as blood pressure and various diabetes measures) with 50% (2/4) of studies reporting a positive change in clinical measure [9]. Goldzweig et al found most positive changes with 75% (6/8) of studies in their review reporting a positive change as a result of PAEHR access [15].

Ammenwerth et al found that the impact of PAEHR access on health outcomes is limited with respect to impact on clinical outcome, health resource consumption, patient adherence, and patient-physician communication. They report that the parameters studied did not show a statistically significant difference between intervention and control groups and in particular, no statistically significant changes could be observed for parameters related to clinical outcome. Ammenwerth’s findings suggest that the available evidence does not support the assumption that PAEHRs improve patient care [1].

### Health Care Professionals’ Perspective

**Figure 4:**
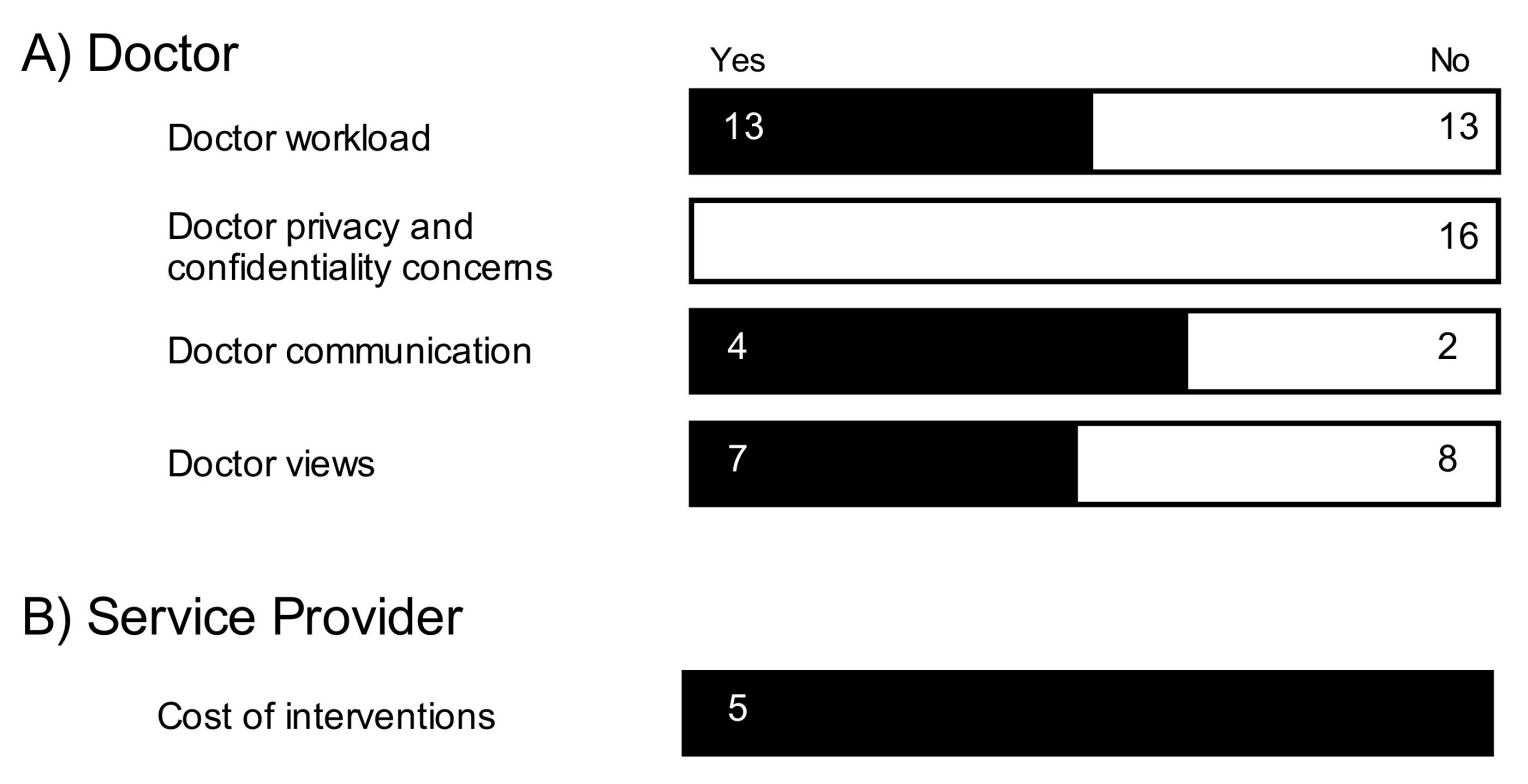
Frequency of studies showing a positive change (black bars) and negative/no change (white bars) after patients were given record access from the point of view of doctors and service providers.

There were a number of articles that evaluated the benefit of PAEHRs from the HCPs’ perspective (Multimedia Appendix 1), although relatively fewer studies focused on the HCPs’ perspective of PAEHRs when compared with patient perspective [20] (Figure 4). The types of HCP evaluated were mainly doctors and nurses [21-30].

Several of these, in addition to stating a qualitative benefit, described the measurable impact of any benefit as outcome measures including workload, privacy and confidentiality concerns, cost, and communication. These are described in more detail below.

#### Doctors’ Views, Privacy, and Confidentiality Concerns

The poor uptake of electronic health records (EHRs) may be driven by HCPs who are wary of patient access to medical records, fearing it may cause patient anxiety. De Lusignan et al found eight studies where physicians feared that PAEHR access without a physician available to interpret the information might cause patients to worry [16]. Although these risks are low [31], doctors have concerns about shared medical records and see less potential for benefit than patients [32]. These concerns included doctors finding a computer system “stressful”, having spent twice as much time using the computer than they had previously using their hand-written notes [19].

These concerns are also extended to the security of the electronic records, with HCPs reporting professional concerns about privacy and confidentiality in 16 studies of de Lusignan’s review [16]. The security and confidentiality of patient data must be put at the forefront of EHR services in order to achieve widespread consumer acceptance and adoption [9], and patients should have the right to decide who can access and edit their medical records [33], which was found to be a common barrier for PAEHR uptake [15].

#### Workload

HCPs do not want changes to the current medical record system to negatively impact their time [34]. Research has shown an interesting mix of findings on the impact of PAEHRs on workload. The most striking finding is a study that recently investigated changes in HCP workload [16]. De Lusignan et al found that half of the studies in their review (13/26) showed PAEHRs have a positive impact on changes to workload or workflow (ie, a decrease in workload).

Poissant et al’s review focused on the effects of PAEHR access on HCPs documentation time. They found that that decreased documentation time in a PAEHR project is not likely to be realized, especially for physicians. From a total of 23 studies included in their review, they found that 11 studies examined the impact of PAEHRs on time efficiencies of nurses, of which six studies found that nurses are more likely than physicians to gain time efficiencies by using a computer system to document patient information. Two studies found that bedside PAEHR increased documentation time, and one study reported different results depending on the specific content of the information being documented [19].

With respect to physicians, ten studies examined the impact of PAEHR on time efficiencies of physicians. Poissant et al found that using a PAEHR system increased physician documentation time by 17%. Of their studies, 60% (6/10) reported significant results in the direction of unfavorable impact on initial visit time, and 10% (1/10) lacked sufficient information to identify whether the results were significant. In the remaining three studies, there were no significant differences between computer and paper documentation time [19]. Ferreira et al report that physicians found no change in their workload or no adverse consequences as a result of PAEHRs, and all the physicians supported the use of PAEHRs [4].

#### Doctor Communication

Improving doctor-patient relationship is one of the few outcomes that can be investigated from a physician point of view, yet studies still report how doctor-patient relationships improve from the patient point of view. Ferreira et al report only one study that investigated doctor-patient relationships. They found that the majority of doctors (and patients) were unanimous in their belief that the paehr access was positive for both physicians and patients and improved the level of communication between them [4]. Furthermore, Ferreira et al report three randomized clinical trial studies whereby hcps found access to paehrs via the internet easy to use, useful, and considered that it could improve their communication with other HCPs [4].

#### Cost for Patient Accessible Electronic Health Records

Fewer studies across the reviews examined PAEHRs from the perspective of the service provider (eg, a hospital providing PAEHR access). In one study, Poissant et al found that using PAEHRs for writing all inpatient orders significantly lowered patient charges and hospital costs [19]. Nyugen et al reviewed three studies that demonstrated how PAEHRs in the United States could provide a positive return on investment providing evidence of major financial benefit [13].

#### Data Quality

Apart from patients, HCPs, and service provider factors, we considered study design, which informs the quality of the evidence analyzed in our review. Poissant et al reviewed 23 papers of which only 5 were randomized controlled trials (RCT), with other studies being posttest control studies (n=6), and one-group pretest-posttest designs (n=12) [19]. Ferreira et al outlined the number of articles implementing an RCT (n=18), a transversal study (n=39), a longitudinal study (n=5), and a letter (n=20) [4].

Not all studies highlighted the historically small proportion of randomized studies. In Giardina et al’s more recent review, however, 20 studies were RCTs with only seven studies being uncontrolled observational studies [9], suggesting that the quality of evidence is continuing to improve.

## Discussion

### Principal Findings

The systematic reviews included in our synthesis aimed to investigate the effect of record access on various outcomes. We found that these reviews showed mixed outcomes in aspects of patient safety, usefulness, satisfaction, and self-efficacy across patients and HCPs. This is typically represented by Giardina et al’s review, who found an absence of positive evidence on these outcome measures, with only 50% of studies showing positive changes with record access [9]. Positively, the little work carried out on the cost of PAEHRs has shown that implementing PAEHR systems would lower hospital costs.

We next highlight some of the issues that surround the study of patient access to their medical records in terms of both technical and scientific rigor, which leads to the root of the problem: for such a large problem, there is very little data-driven evidence coming from a large population. We believe a large factor contributing to the lack of success in PAEHR access has been a lack of data-driven evidence about the opinions, wants, and needs of large clinical consumer groups. This setback comes to the heart of the issue in this field: PAEHR developers are still not clear whether providing patients with record access makes a difference to either the patients themselves or their physician.

### Lack of Empirical and Rigorous Testing

Current research is targeted to certain clinical groups and their needs, which makes the findings difficult to implement across a large non-disease-specific population. More than half of the patient portal evaluations reviewed by Otte-Trojel were targeted at chronic disease patients, such as the management of diabetes, hypertension, and depression [7]. The problem with disease-specific studies is that they are more vulnerable to a “ceiling effect” due to the breadth and quality of the well-established existing disease management programs. This problem is also highlighted in Goldzweig’s review, which identified examples where record access was associated with improved outcomes for patients with chronic diseases, such as diabetes, hypertension, and depression, but these studies generally used the PAEHR in conjunction with case management [15]. As a result, the effects of PAEHRs are small and could provide an explanation into why PAEHR effects are often inconsistent. Future work could consider investigating the effects of PAEHRs on various mechanisms (such as patient empowerment) outside the remit of disease-specific groups to avoid issues surrounding care coordination [7].

A large proportion of studies that investigate the impact of PAEHRs on various outcome measures follow a quasi-experimental design implementing interviews and/or surveys to measure the impact of each intervention. There is the potential to implement better quality study designs and use more objective and rigorous measuring techniques to determine whether a cause and effect relationship exists between PAEHRs and outcome measures. Future research should examine the processes of PAEHR and their direct effects by implementing a pretest and posttest design where participants are tested on a specific set of outcome measures before and after exposure to a PAEHR system.

Research should also aim to address our understanding of how PAEHRs can bridge the gap between patient and doctor with a focus on using up-to-date technologies. Over the last 20 years, there have been large technological improvements, both in terms of hardware and software. As a result, research carried out in the last century may not be comparable with modern day technologies. We found that a large proportion of studies that investigated the effects of PAEHRs were published between 1996 and 2005. The implementation of PAEHRs should no longer be a technological problem as the technology has been available for some years now [13], therefore, it is important for research to reflect these advances.

### Limitations

Our study focused only on English language reviews, which neglects PAEHR advancements from other parts of the world. Furthermore, our review of reviews covers a small overall evidence base compared to a systematic review focused on one group (eg, patients) and a lack of quantitative synthesis is arguable, as the reviews presented heterogeneous datasets/studies. However, we believe that the reviews analyzed here cover a large number of primary studies across a variety of outcome measures and our scoring system provides a quantifiable way of synthesizing the literature. PAEHR systems conceptually vary, and our review brings together results across a variety of PAEHR systems, as do the reviews that make up our work, which could be contributing to the nature of the results. The small number of RCTs investigating patient access to their medical records was further limited by the small sample sizes in the studies used in this review, therefore compromising the quality of a scientific study. However, there is currently little solid evidence from RCTs of proven effectiveness in improved patient health outcomes through the use of PAEHRs [35].

### Conclusions

Our synthesis of available systematic reviews examined the impact of patient access to electronic medical records and revealed few overarching results. There was minimal evidence to support the universal use of PAEHRs both from a patient or HCP point of view; however, PAEHRs appear to have a positive impact on patient empowerment. Patients appear to have positive views after using PAEHRs and the information quality in PAEHRs is positive, although major drawbacks include security, privacy, and confidentiality concerns. HCPs also appear to be divided in terms of whether using a PAEHR reduces their workload. The topic of PAEHRs appears to be one that divides both patients and HCPs and is certainly a field where more rigorous research is needed to evaluate practice and improve system design and implementation.

## Appendix

Multimedia Appendix 1 Definitions of outcome measures across synthesized reviews.

Multimedia Appendix 2 Evidence synthesis overview.

## Abbreviations

EHR: electronic health record
HCP: health care provider
PAEHR: patient accessible electronic health records
RCT: randomized controlled trial
