# Supplementary material for: “Nothing About Me Without Me”: An Interpretative Review of Patient Accessible Electronic Health Records
Source: J Med Internet Res. 2015 Jun 29;17(6):e161. doi: 10.2196/jmir.4446 (PMC4526966; doi:10.2196/jmir.4446)
Supplement: Multimedia Appendix 1 [file jmir_v17i6e161_app1.pdf]

Multimedia Appendix 1. Definitions of outcome measures across synthesized reviews (to keep subjectivity to a low, the definitions in column 1 used in the current paper have been derived from the review definitions in column 2; column 3 provides references to the original definitions).

**Definition**	**Wording used in the original reviews to derive definitions**	**Source**
Usefulness/effectiveness of record access (definition from Giardina)	Effectiveness of record access (e.g. physical health outcomes, psychosocial health outcomes, health behaviours and adherence outcomes)	Giardina
	Usability and usefulness, Interoperobility, Usability, Adoption rate, Documentation practice, Changes to workflow and productivity (with usefulness of record access as a key outcome measure), Amount of time with patients, Quality of clinician-patient interactions, Quality and Safety of Care, Medication Error prevention	Nyugen
	Time saving (e.g. avoiding an in-person clinic visit)	de Lusignan
	Adherence (e.g. adherence to medical advice)	Goldzweig
	Various (e.g. effect of RA use)	Poissant
Information Quality (definition from Nyugen)	Improved Information Quality, Accuracy, Completeness of data, Timely and better access to up-to-date patient information, Availability, Improved legibility, Issues in dealing with data overload and overflow	Nyugen
Self-efficacy (patient empowerment) (definition combined through source studies)	Patient empowerment	Amante
	Patient empowerment (e.g."patients being empowered to communicate more effectively with clinicians")	de Lusignan
Patient satisfaction (definition from Giardina)	Patient satisfaction (Satisfaction questionnaire), Clinical (care) satisfaction	Giardina
	User satisfaction	Nyugen

	Attitudes (based on "health outcomes, health status, satisfaction, or adherence")	Goldzweig
	Level of certainty, mood states, satisfaction with care	Ko
Patient views (definition combined through source study)	Patient views	Giardina
Patient engagement (definition combined through source studies)	Medication modifications, Usage (e.g. PAEHR system logins)	Giardina
	Patient engagement (e.g. via attendence to health checks)	Giardina, Amante, Goldzweig
Clinical outcome (definition combined through source studies)	Glycemic control, Change in GHb, Blood pressure control	Giardina
	Diabetes Recognition Program (DRP) scores	Amante
	health outcomes, health status, satisfaction, or adherence	Goldzweig
	HbA1c	Ko
Work load (Definition from de Lusignan)	Workload (e.g. changes to workload and workflow)	de Lusignan
Self-efficacy (patient involvement) (definition combined through source studies)	Informed patient, Patient involvement in care	Giardina
	patient utilization and engagement data, clinical outcomes	Amante
	Patient involvement	de Lusignan
	Communication, Medication accuracy and safety (as a result of patient's being involved in their care through record access)	Goldzweig
	Global health status, emotional functioning, and cognitive functioning (measured using the European Organization for Research and Treatment of Cancer QLQ-C30 questionnaire)	Ko

	Patient interest Interest in examining their medical record and the reasons for their interest	Ferreira
Psychological (definition combined through source studies)	Anxiety, Quality of life, Anxiety/depression/contentment	Giardina
	health outcomes, health status, satisfaction, or adherence	Goldzweig
	Global health status, emotional functioning, and cognitive functioning (measured using the European Organization for Research and Treatment of Cancer QLQ-C30 questionnaire), Verona Service Satisfaction Scale-54 (VSSS-54) and the Krawiecka and Goldberg (K & G)	Ko
	Anxiety levels	Ferreira
	Stress	Poissant
Doctor views (definition combined through source study)	Doctor's perception	Giardina
	Clinician satisfaction (User satisfaction)	Nyugen
Cost of interventions (definition combined through source studies)	Cost of interventions	Giardina
	Administration efficiency and cost reduction	Nyugen
	To assess the effects on health care resource utilization of a network of microcomputer workstations (record access) for writing all inpatient orders	Poissant
Self-efficacy (communication) (definition combined through source studies)	Information sharing	Giardina
	Attitudes	Goldzweig
Self-reported health outcome/behaviour (definition from Giardina)	Health related behaviours (e.g. diet, smoking, medications)<br>Self reported health outcomes (e.g. physical health functioning)	Giardina

	Subjective views of patient satisfaction with communication and perception of communication as determined by structured interview, Self-reported use of the PAEHR , Health related behaviours	Ko
Security, privacy and confidentiality concerns (definition combined through source studies)	Concerns over privacy and confidentiality	Nyugen
	Privacy and security concerns	de Lusignan
	Attitudes	Goldzweig
