# Supplementary material for: “Nothing About Me Without Me”: An Interpretative Review of Patient Accessible Electronic Health Records
Source: J Med Internet Res. 2015 Jun 29;17(6):e161. doi: 10.2196/jmir.4446 (PMC4526966; doi:10.2196/jmir.4446)
Supplement: Multimedia Appendix 2 [file jmir_v17i6e161_app2.pdf]

Multimedia Appendix 2. Evidence synthesis overview – including article details, and synopsis of each included review.

Study number (Reference)	Authors	Title	Year	Journal	Years of studies included	Number of studies in review	Patient or clinician oriented studies	Geography (countries covered in review)	Primary aim	Findings
1 (11)	Giardini	Patient access to medical records and healthcare outcomes: a systematic review	2014	Journal of the American Medical Informatics Association	1977-2012	27	Patient	USA & UK	To investigate the effect of RA on (1) measures of safety, (2) effectiveness (3) patient-centeredness (4) timeliness (5) efficiency (6) equity	(1) Mixed outcomes, with 50% of studies showing positive changes with RA, (2) RA appears to increase patients perceptions of control
2 (13)	Nguyen	Electronic health records implementation: An evaluation of information system impact and contingency factors	2014	International Journal of Medical Informatics	2001-2011	98	Patient	Mixed **	To review EHR benefits and issues associated with EHR implementation.	(1) A mix of positive and negative impacts of EHR was found across different evaluation dimensions. (2) A number of contingent factors were found to contribute to successful implementation of EHR.
3 (35)	Amante	A Systematic Review of Electronic Portal Usage Among Patients with Diabetes	2014	Diabetes, Technology & Therapeutics	2005-2014	16	Patient	USA	(1) Examine characteristics associated with enrollment and utilization of portals among patients with diabetes; (2) Identify barriers and facilitators of electronic patient portal enrollment and utilization	Barriers to enrollment included a lack of patient (1) capacity and (2) desire. Barriers to portal utilization included (1) patient capacity and (2) lack of provider and patient buy-in to portal benefits. Facilitators included provider and family recommendation.
4 (16)	de Lusignan	Patients' online access to their electronic health records and linked online services: a systematic interpretative review	2014	BMJ Open	Not specified	143	Both	Mixed	To identify and understand (1) barriers and facilitators for providing online RA; and (2) their association with organisational/IT system issues.	(1) Patient online access and services offer increased convenience and satisfaction. However (2) professionals were concerned about impact on workload and risk to privacy.

5 (7)	Otte-Trojel §	How outcomes are achieved through patient portals: a realist review	2014	Journal of the American Medical Informatics Association	2003 - 2014	32	Patient (and service delivery)	USA	To examine how patient portals contribute to health service delivery and patient outcomes. The specific aims were to examine how outcomes are produced, and how variations in outcomes can be explained.	As a complement to existing health services, patient portals can lead to improvements in clinical outcomes, patient behavior, and experiences.
6 (15)	Goldzweig	Electronic Patient Portals: Evidence on Health Outcomes, Satisfaction, Efficiency, and Attitudes	2013	Annals of Internal Medicine	1990-2013	46	Patient	USA	To review the literature reporting the effect of patient portals on clinical care.	(1) Currently insuffiecient evidence about the effect of patient portals on health outcomes.
7 (1)	Ammenwerth §	The Impact of Electronic Patient Portals on Patient Care: A Systematic Review of Controlled Trials	2012	Journal of Medical Internet Research	1990-2011	5	Patient	USA & Netherlands	To systematically review the available evidence on the impact of electronic patient portals on patient care	The available evidence does not support the assumption that EHRs empower patients and improve patient care.
8 (14)	Ko	Patient-held medical records for patients with chronic disease: a systematic review	2010	Quality & Safety in Health Care	1980-2009	14	Largely patient	Mixed ***	Does a PAEHR improve clinical care, patient outcomes or satisfaction in chronic disease patients	There is no clear benefit of implementing a PAEHR
9 (4)	Ferreira	Why facilitate patient access to medical records	2007	Studies in Health Technology and informatics	1990-2005	14	Largely patient (few studies mentioning clinicians)	Unclassified	To systematically review the potential benefits and drawbacks on (1) patient attitudes and (2) doctor-patient relationships on medical practice.	RA improved patients' (1) education, (2) communication and (3) self-efficacy in their treatment, but (4) patients find the notes difficult to understand and (5) are worried about security, although patients are reassured by technological advances
10 (19)	Poissant	The Impact of Electronic Health Records on Time Efficiency of Physicians and Nurses: A Systematic Review	2005	Journal of the American Medical Informatics Association	1984-2004	23	Clinicians (and nurses)	Unclassified	To estimate the extent of which an EHR affects clinicians' documentation time and identify factors that may explain effiency differences between studies	(1) Decreased documentation time in an EHR project is not likely to be realized, especially for clinicians (2) Nurses are more likely than physicians to gain time efficiencies by using a computer system to document patient information.

** USA = 62; Denmark and England = 5; Norway = 4; Canada = 3; Australia, Holland, Ireland and Israel = 2; Austria, Cyprus, France, Serbia, Sweden, Japan, Korea, Kuwait, Cameron, Uganda = 1; Cross-country: 1 (Sweden and USA)									
*** England = 5; Netherlands = 4; Canada = 1; New Zealand = 1; Scotland = 1; Wales = 1									
§ Excluded reviews from the scoring system analysis									
